# Supplementary material for: Towards epitaxial graphene p-n junctions as electrically programmable quantum resistance standards
Source: Sci Rep. 2018 Oct 9;8:15018. doi: 10.1038/s41598-018-33466-z (PMC6177418; doi:10.1038/s41598-018-33466-z)
Supplement: Supplementary file 1 — Supplementary Information [file 41598_2018_33466_MOESM1_ESM.pdf]

Supplementary information: Towards epitaxial graphene *p-n* junctions as electrically programmable quantum resistance standards

Jiuning Hu[1,2*], Albert F. Rigosi[1*], Mattias Kruskopf[1,2], Yanfei Yang[1,2], Bi-Yi Wu[1,3], Jifa Tian[1,4], Alireza R. Panna[1], Hsin-Yen Lee[1,5], Shamith U. Payagala[1], George R. Jones[1], Marlin E. Kraft[1], Dean G. Jarrett[1], Kenji Watanabe[6], Takashi Taniguchi[6], Randolph E. Elmquist[1], and David B. Newell[1]

[1]Physical Measurement Laboratory, National Institute of Standards and Technology (NIST), Gaithersburg, MD 20899, USA
[2]Joint Quantum Institute, University of Maryland, College Park, MD 20742, USA
[3]Graduate Institute of Applied Physics, National Taiwan University, Taipei 10617, Taiwan
[4]Department of Physics and Astronomy, and Birck Nanotechnology Center, Purdue University, West Lafayette, Indiana 47907, USA
[5]Theiss Research, La Jolla, CA 92037, USA
[6]National Institute for Materials Science, 1-1 Namiki, Tsukuba 305-0044, Japan
*hujiuning@gmail.com, albert.rigosi@nist.gov

Contents

1. Different values of R_{xx} as a function of two gate voltages and B-field
2. Dirac point determination for G1 and G2

1. Different values of R_{xx} as a function of two gate voltages and B-field

At the end of this document, we provide the dependence of the R_{xx} maps as a function of two gate voltages. The maps are presented in successive rows of panels to show their dependence on the magnetic field and are arranged from left to right: UE (R_{xx}), LE (R_{xx}), UE (R_{xy}), LE (R_{xy}).

2. Dirac point determination for G1 and G2

To verify the expected symmetry seen between Figure 4 (a) and (c) in the main text, and to have a rigorous knowledge of the carrier density for any applied top gate voltages, the Dirac point was determined for both G1 and G2.

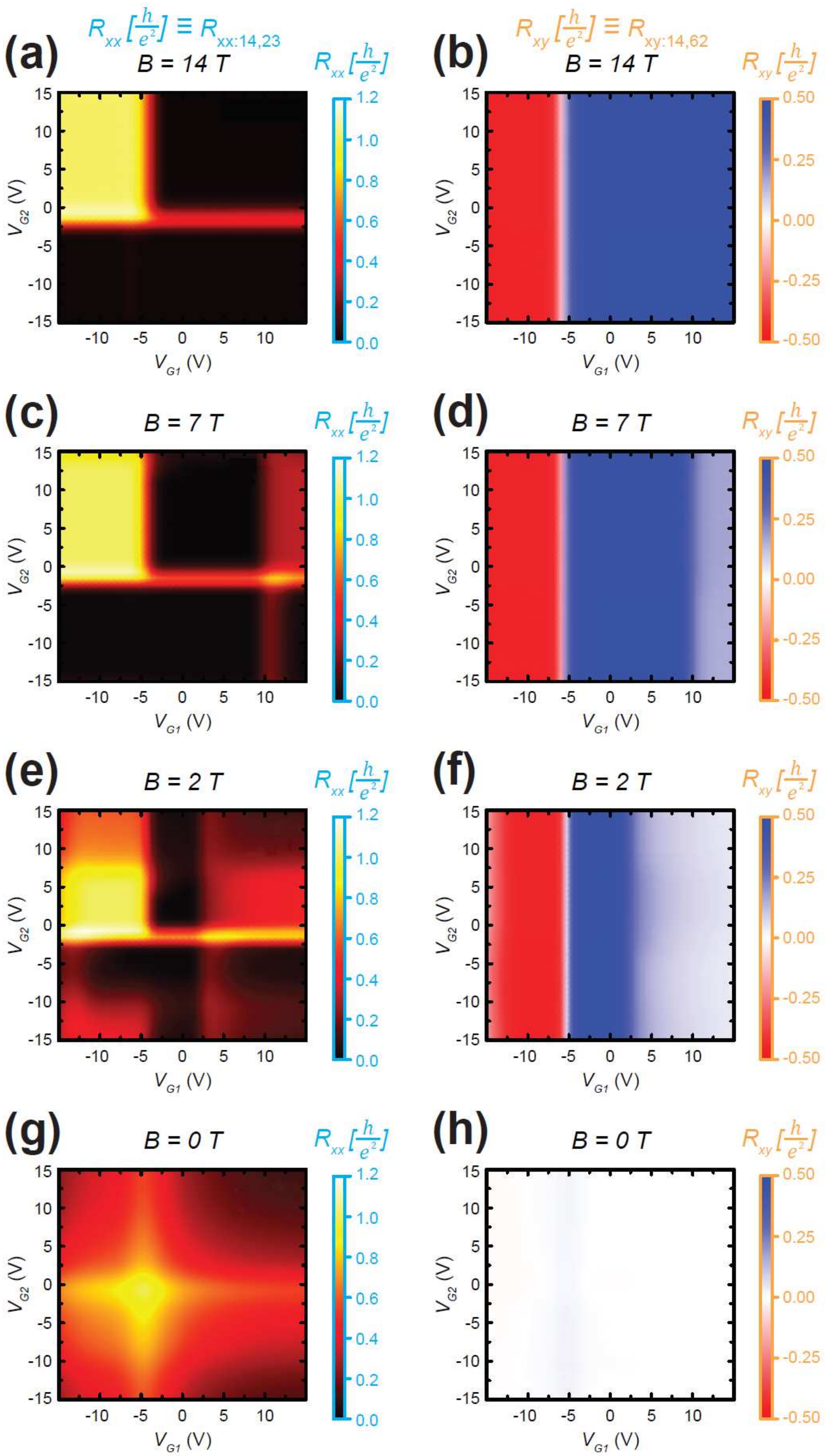

$R_{xx}\,[\frac{h}{e^2}] \equiv R_{xx:14,23}$
$R_{xy}\,[\frac{h}{e^2}] \equiv R_{xy:14,62}$
(a) B = 14 T
(b) B = 14 T
(c) B = 7 T
(d) B = 7 T
(e) B = 2 T
(f) B = 2 T
(g) B = 0 T
(h) B = 0 T
V_{G1} (V)
V_{G2} (V)
$R_{xx}\,[\frac{h}{e^2}]$
$R_{xy}\,[\frac{h}{e^2}]$

Figure 1-SM. The longitudinal and Hall resistances are displayed side-by-side with decreasing magnetic field towards the bottom of the figure. (a) and (b) are data collected at 14 T. The magnetic field in the subsequent panels are: (c) and (d) 7 T, (e) and (f) 2 T, and (g) and (h) 0 T.

We include a few representative panels of how the B-field affects the resistance maps in Figure 1-SM. The Dirac points in Figure 1-SM are determined by horizontal or vertical cuts in the R_{xx} maps, and for positive B fields, the results are averaged to obtain the final Dirac points. The off-diagonal cut across the Dirac points for the R_{xx} maps at different B fields are plotted in Figure 3 in the main text.

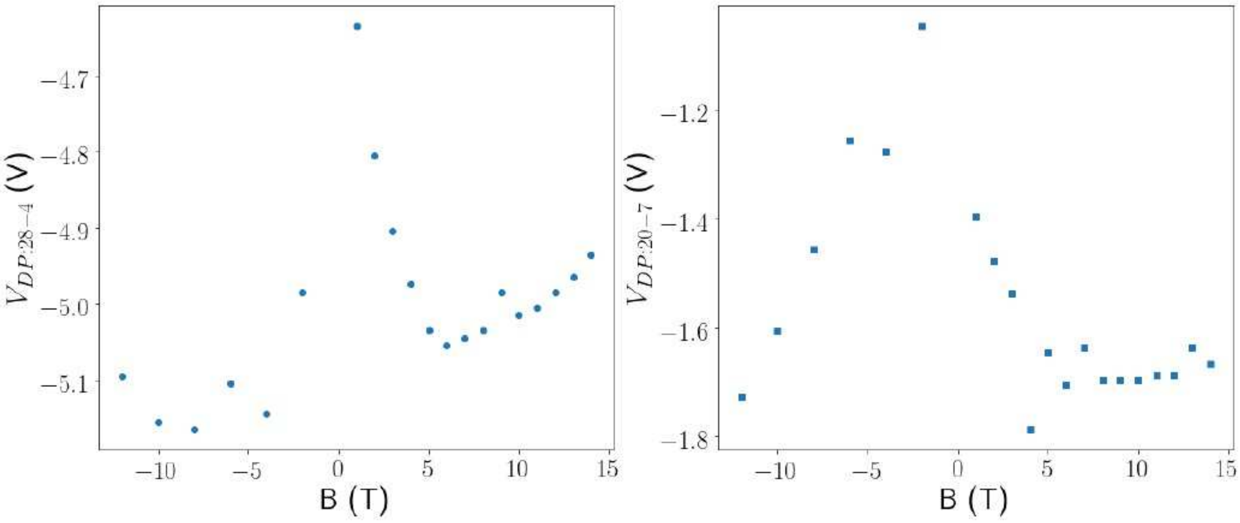

Figure 2-SM. (left) The data for the Dirac point of G1 are acquired at 1.7 K as a function of magnetic field, with the corresponding charge neutrality point being approximately at -1.68 V. (right) The Dirac point of G2 is determined with the same procedure, yielding the approximate charge neutrality point of -5.01 V.

After gaining the knowledge of the two Dirac points shown in Figure 2-SM, the expected points of symmetry were identified and marked in Figure 6 of the main text.
